# Effects of vitamin D supplementation under different salinities on hybrid red tilapia growth performance, hormonal profile and growth genes expression

**DOI:** 10.1038/s41598-025-20669-4

**Published:** 2025-10-02

**Authors:** Hassan H. Silem, Amal M. El-Nahla, Ahmed G. A. Gewida, A. Y. Badawy, Monay M. Shahin, Mohamed F. Badran

**Affiliations:** 1 Aquatic Hatchery Production Department , Fish farming and Technology Institute, Suez Canal University , Ismailia, 41522 Egypt; 2 Physiology Department, Faculty of Veterinary medicine, Suez Canal University, Ismailia, 41522 Egypt; 3 Fish Production Department, Faculty of Agriculture, Al-Azhar University, Cairo, Egypt; 4 Animal Production and Fish resources Department, Faculty of Agriculture, Suez Canal University, Ismailia, 41522 Egypt

**Keywords:** Red tilapia, Salinity, Vitamin D, Growth performance, Gene expression, Aquaculture, Animal biotechnology, Animal physiology, Ichthyology

## Abstract

This study investigated the effects of salinity and vitamin D (VD) supplementation on the physiological and molecular responses of red tilapia hybrid juvenile. The research comprehensively examined growth performance, hormonal profiles, and gene expression under different experimental conditions. Growth performance analysis revealed significant improvements with increasing salinity levels and VD supplementation. The highest growth parameters were observed at 30 ppt salinity with vitamin D3 supplementation, including peak final weight (42.04 g), maximum average daily gain (0.467 g), and optimal feed conversion ratio (0.927–0.967). Survival rates consistently improved, reaching 84.33% under optimal conditions. Hormonal parameters demonstrated notable modulation, with growth hormone (GH) levels showing marked increases, particularly at 15–30 ppt salinity. Adrenocorticotropic hormone (ACTH) remained relatively stable across treatments, suggesting minimal stress response. Molecular analysis of gene expression revealed complex interactions between salinity and VD. The growth hormone (GH) gene showed significant upregulation, particularly at 30 ppt salinity with VD supplementation. The insulin-like growth factor II (IGF-II) gene expression exhibited a non-linear response, with substantial upregulation at 0 ppt salinity and downregulation at 30 ppt salinity. These findings highlight the intricate physiological adaptations and the role of vitamin D in juveniles of red tilapia hybrid under different salinity levels, Therefore, it is advisable to use 0.8 mg of VD followed by 0.4 mg under different salinity levels to enhance the growth performance, feed utilization, GH level, and expression of GH and IGF-II genes. Additionally, culturing fish at 30 ppt salinity followed by 15 ppt appears to improve overall performance compared to freshwater culture.

## Introduction

Teleost fish growth and development occur following species-specific processes and are directly influenced by environmental factors. Fish rely on both external (ecological) and internal (nervous, endocrinological, and neuroendocrine) factors to regulate or synchronize a variety of activities or functions, including their ability to grow^1,2^. Salinity is a critical environmental factor that impacts fish production and can significantly influence various physiological processes in fish. Maintaining salinity within the optimal range for a given fish species is important for maximizing growth, feed utilization, and overall yield production^3,4^. Salinity changes can lead to numerous metabolic adjustments in fish. Furthermore, It can influence food intake and feed conversion efficiency, while optimal salinity levels tend to optimize food consumption leading to improved feed conversion ratios^1,3^. Salinity changes can affect hormonal stimulation, metabolism and biochemical analysis in fish, which in turn impacts growth, development, osmoregulation and reproduction - key factors in fish production^5,6^.

Extreme salinity can increase the energetic cost of osmoregulation and ion transport, causing fish to consume more energy reserves ratios^1,3^. Increased salinity for some fish species can negatively impact growth performance, survival, and lead to skeletal deformities due to the increased energy demands for osmoregulation in hyperosmotic environments^7^. Analysis of rainbow trout (*Oncorhynchus mykiss*) showed that salinity acclimation can affect the osteoblast and osteoclast activity, as well as the scales’ phosphorus and calcium content and led to differential expression of microRNAs involved in bone metabolism pathways like MAPK, calcium signaling, WNT signaling, and mineral absorption^8^.

On the other hand, dietary additives are new immune-stimulant tools for aquatic animals, and in some cases, it can enhance growth, survival ratios reproduction, alleviate diseases and diminish stress effects; the dietary nutrient additions used are anticipated to be safe, economical, eco-friendly, and non-toxic^9,10^. Nutrients can be classified^10^ into, macronutrients include proteins, fiber, carbohydrates and fats, and dietary micronutrient additives (e.g. vitamins and minerals). One significant fat-soluble vitamin is vitamin D (VD). There are two natural forms of VD; cholecalciferol (VD3) and ergocalciferol (VD2), and the primary form in animals is VD3^11,12^. Besides its VD function in normalizing the homeostasis of phosphorus and calcium, VD3 also plays an important part in a wide range of physiological functions equivalently in all animals including aquatics^13,14^. VD promotes fish growth and biomass production by improving feed efficiency and weight gain when supplemented at optimal levels^15,16^. VD plays a novel and crucial role in osmoregulation by enhancing ionocyte differentiation and increasing the number of ion-transport cells in the skin and gills of fish. It regulates acid secretion and the expression of ion transporters, which maintain acid-base balance and ionic homeostasis under environmental stress such as salinity levels^17,18^.

Tilapia aquaculture is globally significant due to its rapid growth rate in freshwater environments, high yield, flavor-some flesh and elevated disease resistance. Furthermore, it strongly tolerates environmental stressors and endures both natural and artificial feed^19,20^. Though it is mainly a freshwater fish, many species of tilapia are euryhaline and can be cultured in fresh, brackish, or even marine water^21^. The hybrid red tilapia, a genetically modified strain of tilapia developed mainly for aquaculture by crossing different species of the genus *Oreochromis*, has achieved widespread acceptance due to its rapid growth rate, salinity tolerance, and adaptability to diverse cultivation environments^22,23^. These hybrids comprise several strains, including Taiwanese red tilapia, Florida red tilapia, and Malay red tilapia^24^. Some crosses, like Taiwanese and Florida red tilapia, inherit increased salinity tolerance and can survive and grow in water with salinities up to full seawater, whereas Nile tilapia typically cannot withstand salinity above 20 ppt^20,25^. As of 2022, global tilapia production was estimated at approximately 6.5 million metric tons (mt), with around 68.9% of the production accounted for as hybrid red tilapia, while the total production of tilapia species in Egypt was about 1,130,430 mt, including hybrid red tilapia^26–29^. Hybrid red tilapia has a rapid growth rate and salinity tolerance, which made it one of the promising species for farming in Egypt, which faces a shortage of freshwater for aquaculture because of various factors, including limitations of freshwater resources, the increasing demand for water for various purposes, and climate change^20,30,31^. In this context and given the role of vitamin D in the growth, osmoregulation process and resistance to environmental stressors such as salinity, the present study aimed to evaluate the effects of different levels of vitamin D under three water salinities on the growth performance, hormonal profiles, and growth-related gene expression of juvenile hybrid red tilapia.

## Materials and methods

### Larvae collection and maintenance

A total of 2700 larvae of the Florida red tilapia hybrid (0.2 ± 0.03 g) were used in this study. Only healthy and disease-free larvae were collected for the experiment. The larvae were purchased from Hosny Tahon hatchery at Kafr El-Shaikh and transported by plastic bags supplied with oxygen to the FFTI, SCU, Ismailia Governorate, Egypt.

Prior to the experiment, the larvae were acclimated for approximately two weeks to the experimental conditions. They were stocked in 3 m^3^ indoor fiber rounded tanks filled with 0, 15, 30 ppt water salinity under controlled water temperature and artificial lighting. The water was purified using sandy filters and UV sterilizers. Vigorous aeration was provided by an air-blower through diffuser air stones.

### Water physio-chemical parameters

The tanks were continuously supplied with aerated water from overhead reservoirs. Water quality parameters were measured daily and maintained within the following ranges: water temperature: 28 ± 1° C, salinity: 0, 15, 30 ppt, pH: 7.60 ± 0.64, dissolved oxygen: 7.3 ± 1.22 mg/L. Additionally, 25% of the water was replaced daily through siphoning. Salinity was measured by digital refractometer (DRBS-300), water temperature and pH by using (Milwaukee MW-100) and DO by using (ExStik II D-0600, FLIR Systems, USA).

### Feeding protocol

Daily feed intake was determined based on the number and weight of the fish as well as water temperature. The fries were fed at a 5–8% rate three times daily for 90 days, with periodic adjustments to the feeding rate at 10-day intervals. The powder and pelleted feed were purchased from Skretting Egypt for Animal Nutrition Factory, EL Asher of Ramadan, Egypt (30.4% crude protein, 3.46% fiber, 6.26% lipids, 450 kcal/100 g gross energy, and 66.45 mg CP: Kcal, 0.03 mg VD/kg). Vitamin D3 purchased from Karma care Co., Egypt, was incorporated into the feed in two levels (0.4 & 0.8 mg/kg) via spraying and vacuum coating as reported by^23,32,33^. The VD was added to raise the concentration from 0.03 mg in the basal diet to 0.4 and 0.8 mg in the treated feeds. Each prepared diet was individually sealed in a clean, dry plastic bag, boxed, and stored at 4 °C until use.

### Experimental design

All larvae were divided into 9 groups with each group having 3 replicated. They were distributed into 27 tanks (100 fish for each) as follows: -.

**Table:** 

Treatment (Factorial)		
	Salinity	Vitamin D3
T_1_	0 ppt	D_0_ (0.03 mg/kg)
T_2_		D_1_ (0.4 mg/kg)
T_3_		D_2_ (0.8 mg/kg)
T_4_	15 ppt	D_0_ (0.03 mg/kg)
T_5_		D_1_ (0.4 mg/kg)
T_6_		D_2_ (0.8 mg/kg)
T_7_	30 ppt	D_0_ (0.03 mg/kg)
T_8_		D_1_ (0.4 mg/kg)
T_9_		D_2_ (0.8 mg/kg)

### Determination of growth performance

On day 90, the fish were caught, counted, weighed, and measured for length. Growth performance and feed utilization parameters—including initial body weight (**W**_**I**_), final body weight (**W**_**F**_), final total length (**TL**_**F**_), weight gain (**W**_**G**_), total length gain (**TL**_**G**_), condition factor (K), average daily gain (ADG), feed conversion ratio (FCR), protein efficiency ratio (PER), specific growth rate (**SGR**) and survival rate (SR) were calculated for each replicate according to the following Eqs^20,23,32^. :

Weight gain, g (W_G_) = Average final weight – Average initial weight.

Final length gain (TL_G_) = Final total length (cm) − Initial total length (cm).

K=$$\:\frac{Final\:body\:weight\times\:100}{Final\:{total\:length}^{3}}$$

Average daily gain (g/fish/day): ADG= $\:\frac{Final\:body\:weight-Initial\:body\:weight\:}{experimental\:periods}$.

Feed conversion ratio (FCR)= $\:\frac{Feed\:intake\:\left(g\right)}{Total\:weight\:gain\:\left(g\right)}$.

Protein efficiency ratio (PER) = $\:\frac{Weight\:gain\:\left(g\right)}{Protein\:intake\:\left(g\right)}$.

Specific growth rate (%/day): SGR =($\:\frac{Ln.final\:body\:weight-Ln.initial\:body\:weight}{period\:\left(days\right)}$)$\:\:\times\:100$.

Survival rate (SR) (%) = $\:\frac{Final\:stocking\:density}{Initial\:stocking\:density}$ × 100.

### Hormonal profile analyses

Blood samples were collected on day 90 from three fish per replicate. Following anesthesia induced by diluted MS-222 (100 mg/l), blood was withdrawn via cardiac puncture and placed into clean, dry Eppendorf tubes. The samples were allowed to clot, then centrifuged at 5000 rpm for 5 min to obtain the serum^23,34^. The serum was subsequently used to determine hormonal profiles, including Growth hormone (GH) and Adrenocorticotropic hormone (ACTH) using immunoassay analyser (Siemens- Immulite-2000/xpi, Siemens co. Germany).

### Gene expression procedure

#### Samples

On the 90-day, a total of 27 samples/fish from 9 treatments, 3 replicates for each treatment were collected, transferred to the lab and used for gene expression studies.

#### RNA extraction, RNA reverse transcription, and PCR amplification

To obtain the mRNA of the studied genes, 27 samples were used. RNA from tissue samples was isolated using an efficient system for purification of total RNA from tissue, GeneJET RNA Purification Kit^®^ (Catalog #: K0732). The kit utilizes a silica-based membrane technology in the form of a convenient spin column, eliminating the need for tedious cesium chloride gradients, alcohol precipitation or toxic phenolchloroform extractions. RNA molecules longer than 200 nucleotides can be isolated with the GeneJET RNA Purification Kit in 15 min after the lysis step. The high-quality purified RNA can be used in a wide range of downstream applications. The original tubes were kept frozen at − 80 °C until use. Reverse transcription of RNA to cDNA was performed using the protocol of the Maxima H Minus Double-Stranded cDNA Synthesis Kit ^®^ (Catalog #: K2561). The Maxima™ H Minus Double-Stranded cDNA Synthesis Kit is a complete system for efficient synthesis of double-stranded cDNA from total RNA or mRNA. First- and second strand cDNA synthesis reactions are performed in the same tube without the need for intermediate organic extraction or ethanol precipitation steps. This convenient one-tube format speeds up the synthesis procedure and maximizes cDNA recovery. The kit contains premixed components to reduce the number of pipetting steps necessary to complete the procedure.

#### Housekeeping genes

To normalize changes in the studied genes [Growth hormone (GH) and Insulin-like growth factor II (IGF-II)], β-actin was used as the expression reference gene (Table 1). Real- Time PCR was used to analyze the house keeping gene expression.

**Table 1:** Primers used in real-time PCR.

Gene	Primer sequence	Tm	Sequence reference
β-actin	F: CCACACAGTGCCCATCTACGA R: CCACGCTCTGTCAGGATCTTCA	58	NCBI: NM_001009784.2
GH	F: CCAGCCATGAACTCAGGTAAGACA R: TGCTGAGAGGAGACGCCCAAACA	62.7 64.2	M97765
IGF-II	F: TCCCCAGCTGGAAGATGTGTCACG R: CTGGACGCAGCTGAAATCCTGTGG	61.4 54.5	AF033802

#### Delta-delta Ct (2^–∆∆Ct^) method

To calculate the relative fold gene expression of samples by performing real-time polymerase chain reaction (qPCR), 2^–∆∆Ct^ method was used^35^.

ΔΔ*C*_T_ = (*C*_T, Target_ – *C*_, Actin_)_Time x_ – (*C*_T, Target_ – *C*_,Actin_)_Time 0_.

or to simplifying the equation:

ΔΔ*C*_T_ = Δ*C*_T_ (Treated sample) - Δ*C*_T_ (Control sample) where,

Δ*C*_T_ = is the difference between Ct* values gene of interest and housekeeping gene for a given sample. This is to essentially normalize the gene of interest to a gene which is not affected by the experiment, hence the housekeeping gene-term.

Δ*C*_T_ = Ct (gene of interest) – Ct (housekeeping gene).

* Ct (Cycle threshold): Refers to the number of cycles needed for the fluorescent signal to cross the detection threshold during qPCR. A lower Ct value indicates higher initial quantities of the target gene.

### Statistical analysis

Qualitative statistics were initially computed, comprising the mean and standard error (mean ± S.E.) for each parameter. Then results were subjected to Statistical analyses which were carried out using SPSS 25.0. A two-way analysis of variance (ANOVA) incorporating interaction effects examined the influence of distinct treatment groups (D_0_, D_1_, and D_2_) and salinity (0 ppt, 15ppt and 30 ppt), as well as their interactions. Duncan’s multiple-range tests (DMRTs) were employed to compare treatment and concentration groups. Data are presented as means $\:\pm\:$ standard error, with statistical significance established as $\:P<0.05$. Mean comparisons were conducted using LSD according to^36^.

#### Statistical model

**Y**_**ijk**_ **= µ + T**_**i**_ **+ V**_**j**_ **+ e**_**ijk**_ where.

**Y**_**ijk**_: is the observation of the ijk^th^ individual; µ: an underlying overall least squares mean specific to each trait; **T**_**i** =_ the fixed effect of the i^th^ salinity; **V**_**j**_ = the fixed effect of the j^th^ Vitamin D level; **e**_**ijk=**_ the error.

## Results

### Growth performance

#### The effect of vitamin D supplementation under different salinities on growth performance in juvenile hybrid red tilapia

Data presented in Table 2 show that at salinity 15 and 30 ppt significantly (*P* < 0.05) increased the final weight, weight gain, final length, length gain and k factor when compared to 0 ppt. The addition of vitamin D at a dose of 0.08 mg/kg diet also significantly (*P* < 0.05) increases the of final weight, weight gain, final length, length gain and k factor when compared to both D0 (0.03 mg/kg) and D1 (0.4 mg/kg).

Regarding the interaction between salinity and vitamin D, T_6_, T_9_, which correspond to 15, 30 ppt salinity with 0.8 mg of VD exhibited the highest values of final body-weight and weight gain than all groups followed by T_8_ (30 ppt and 0.4 mg of VD) then T_7_ (30 ppt and 0.0 mg of VD). T_9_ (30 ppt and 0.8 mg of VD) could increase the final total length and length-gain significantly, followed by T_4_, T_5_, T_6_, T_7_, T_8_. The K factor value increased significantly on T_6_ while the lowest value was observed on T_1_ (Table 2).

**Table 2:** Qualitative statistical analysis (Mean ± SE) for the effect of salinity and vitamin D on final body-weight, weight gain, final total length, length gain and K factor of juvenile red tilapia hybrids.

Treatments			Final weight (g)	Weight gain (g)	Final length (cm)	Length gain (cm)	K factor
Main effects							
Salinity level							
0 ppt			19.63$\:\pm\:$1.01^b^	19.4$\:\pm\:$1.01^b^	12.87$\:\pm\:$0.17^b^	11.47$\:\pm\:$0.168^b^	0.92$\:\pm\:$0.04^b^
15 ppt			30.87$\:\pm\:$2.22^a^	30.79$\:\pm\:$2.19^a^	13.82$\:\pm\:$0.11^a^	12.42$\:\pm\:$0.11^a^	1.17$\:\pm\:$0.09^a^
30 ppt			34.37$\:\pm\:$2.09^a^	34.17$\:\pm\:$2.09^a^	14.05$\:\pm\:$0.18^a^	12.66$\:\pm\:$0.18^a^	1.23$\:\pm\:$0.06^a^
Vitamin D							
D0			23.67$\:\pm\:1.85$^b^	23.58$\:\pm\:1.87\:$^b^	13.39$\:\pm\:0.22\:$^b^	11.99$\:\pm\:0.22\:$^b^	0.97$\:\pm\:0.06\:$^b^
D1			26.24$\:\pm\:$2.01^b^	26.04$\:\pm\:$2.02^b^	13.47$\:\pm\:0.21\:\:$^b^	12.07$\:\pm\:$0.21^b^	1.06$\:\pm\:$0.05^b^
D2			34.98$\:\pm\:2.98\:$^a^	34.78$\:\pm\:2.98\:$^a^	13.89$\:\pm\:$0.24 ^a^	12.9$\:\pm\:0.24\:$^a^	1.29$\:\pm\:0.08\:$^a^
Two-way ANOVA							
Salinity level			< 0.001	< 0.001	< 0.001	< 0.001	< 0.001
Vitamin D			< 0.001	< 0.001	0.044	0.044	< 0.001
Salinity * VD			0.004	0.005	0.034	0.034	0.011
Interaction							
	Salinity	VD					
T1	0 ppt	D0	16.65$\:\pm\:$0.19^f^	16.45$\:\pm\:$0.19^f^	12.67$\:\pm\:$0.18^c^	11.27$\:\pm\:$0.18^c^	0.82$\:\pm\:$0.04^e^
T2		D1	18.89$\:\pm\:$0.52^f^	18.69$\:\pm\:$0.52^f^	12.77$\:\pm\:$0.43^c^	11.37$\:\pm\:$0.43^c^	0.92$\:\pm\:$0.07^de^
T3		D2	23.37$\:\pm\:$0.52^e^	23.17$\:\pm\:$0.52^e^	13.17$\:\pm\:$0.23^bc^	11.77$\:\pm\:$0.23^bc^	1.03$\:\pm\:$0.07^cde^
T4	15 ppt	D0	25.48$\:\pm\:$0.45^de^	25.62$\:\pm\:$0.72^de^	13.87$\:\pm\:$0.37^b^	12.47$\:\pm\:$0.37^b^	0.96$\:\pm\:$0.08^de^
T5		D1	27.62$\:\pm\:$1.27^cd^	27.42$\:\pm\:$1.27^cd^	13.77$\:\pm\:$0.09^b^	12.37$\:\pm\:$0.09^b^	1.06$\:\pm\:$0.03^cd^
T6		D2	39.52$\:\pm\:$0.41^a^	39.32$\:\pm\:$0.41^a^	13.83$\:\pm\:$0.067^b^	12.43$\:\pm\:$0.07^b^	1.49$\:\pm\:$0.03^a^
T7	30 ppt	D0	28.88$\:\pm\:$0.97^C^	28.68$\:\pm\:$0.97^c^	13.63$\:\pm\:$0.12^b^	12.23$\:\pm\:$0.12^b^	1.14$\:\pm\:$0.07^bcd^
T8		D1	32.20$\:\pm\:$1.11^b^	32.00$\:\pm\:$1.11^b^	13.87$\:\pm\:$0.07^b^	12.47$\:\pm\:$0.07^b^	1.21$\:\pm\:$0.04^bc^
T9		D2	42.04$\:\pm\:$1.88^a^	41.84$\:\pm\:$1.88^a^	14.67$\:\pm\:$0.29^a^	13.27$\:\pm\:$0.29^a^	1.34$\:\pm\:$0.15^ab^

The data is presented as mean $\:\pm\:$ standard error of mean (SEM) in tables.

#### The effect of vitamin D supplementation under different salinities on feed utilizing and survival in juvenile hybrid red tilapia

Regarding salinity levels, 30 ppt followed by 15 ppt could significantly enhance the values of ADG, FCR, PER, SGR, and survival in compared to 0 ppt. While D2 (0.8 mg/kg) exhibited higher significance values, followed by D1 in comparison to D0 (Table 3).

As the interaction results showed in Table 3 and 30 ppt and 0.8 mg of VD (T_9_), significantly increased the values of ADG and SGR followed by T_6_ (15 ppt and 0.4 mg of VD). while 0.4 and 0.8 mg of VD at 15 and 30 ppt enhanced the values of FCR and PER significantly. The highest survival rate was recorded in T_6_ (15 ppt and 0.8 mg of VD) while 0.4 and 0.8 mg of VD could enhance the survival rates under the different salinities (Table 3).

**Table 3:** Qualitative statistical analysis (Mean ± SE) for the effect of vitamin D supplementation under different salinities on average daily gain (ADG), feed conversion ratio (FCR), protein efficiency ratio (PER), specific growth rate (SGR), survival rate (SR) in juvenile red tilapia hybrid.

Treatments			ADG	FCR	PER	SGR		Survival
Main effects								
Salinity level								
0 ppt			0.22$\:\pm\:$0.01^c^	1.42$\:\pm\:$0.09^a^	1.73$\:\pm\:$0.09 ^b^	2.21$\:\pm\:$0.03 ^b^		74.70$\:\pm\:$1.93 ^b^
15 ppt			0.34$\:\pm\:$0.03^b^	1.10$\:\pm\:$0.08^b^	2.24$\:\pm\:$0.15 ^a^	2.55$\:\pm\:$0.03^a^		81.44$\:\pm\:$1.13 ^a^
30 ppt			0.38$\:\pm\:$0.02^a^	1.08$\:\pm\:$0.07^b^	2.28$\:\pm\:$0.13 ^a^	2.48$\:\pm\:$0.03 ^a^		80.21$\:\pm\:$1.27 ^a^
Vitamin D								
D0			0.26$\:\pm\:0.02\:$^b^	1.49$\:\pm\:0.07\:$^a^	1.62$\:\pm\:0.07\:$^b^	2.29$\:\pm\:0.04\:$^b^		76.14$\:\pm\:$1.66^a^
D1			0.29$\:\pm\:0.02\:$^b^	1.06$\:\pm\:$0.06^b^	2.32$\:\pm\:$0.13 ^a^	2.34$\:\pm\:$0.04 ^b^		79.84$\:\pm\:$1.94^a^
D2			0.39$\:\pm\:$0.03^a^	1.04$\:\pm\:0.04\:$^b^	2.31$\:\pm\:0.09\:$^a^	2.48$\:\pm\:0.05\:$^a^		80.37$\:\pm\:$1.43^a^
Two-way ANOVA								
Salinity level			< 0.001	< 0.001	< 0.001	< 0.001		0.010
Vitamin D			< 0.001	< 0.001	< 0.001	< 0.001		0.111
Salinity * VD			0.004	0.050	0.028	0.046		0.733
Interaction								
	Salinity	VD						
T1	0 ppt	D0	0.183$\:\pm\:$0.003^i^	1.743$\:\pm\:$0.069^a^	1.37$\:\pm\:$0.057^d^		2.13$\:\pm\:$0.001^g^	71.90$\:\pm\:$3.80
T2		D1	0.210$\:\pm\:$0.006^h^	1.300$\:\pm\:$0.025^bc^	1.84$\:\pm\:$0.034^bc^		2.19$\:\pm\:$0.01^f^	75.53$\:\pm\:$4.13
T3		D2	0.257$\:\pm\:$0.007^g^	1.203$\:\pm\:$0.023^c^	1.98$\:\pm\:$0.03^b^		2.3$\:\pm\:$0.010^f^	76.67$\:\pm\:$2.66
T4	15 ppt	D0	0.280$\:\pm\:$0.006^f^	1.407$\:\pm\:$0.020^b^	1.69$\:\pm\:$0.02^c^		2.34$\:\pm\:$0.01^de^	79.33$\:\pm\:$1.33
T5		D1	0.303$\:\pm\:$0.014^e^	0.947$\:\pm\:$0.468^d^	2.53$\:\pm\:$0.12^a^		2.38$\:\pm\:$0.02^cd^	80.67$\:\pm\:$2.19
T6		D2	0.437$\:\pm\:$0.003^b^	0.953$\:\pm\:$0.044^d^	2.50$\:\pm\:$0.12^a^		2.55$\:\pm\:$0.01^a^	84.33$\:\pm\:$1.45
T7	30 ppt	D0	0.320$\:\pm\:$0.010^d^	1.337$\:\pm\:$0.041^b^	1.79$\:\pm\:$0.06^bc^		2.40$\:\pm\:$0.01^c^	77.20$\:\pm\:$1.47
T8		D1	0.357$\:\pm\:$0.013^C^	0.927$\:\pm\:$0.038^d^	2.59$\:\pm\:$0.11^a^		2.45$\:\pm\:$0.02^b^	83.33$\:\pm\:$2.72
T9		D2	0.467$\:\pm\:$0.022^a^	0.967$\:\pm\:$0.022^d^	2.47$\:\pm\:$0.06^a^		2.58$\:\pm\:$0.02^a^	80.10$\:\pm\:$0.67

The data is presented as mean $\:\pm\:$ standard error of mean (SEM) in tables.

### The effect of vitamin D supplementation under different salinities on hormonal profile in juvenile hybrid red tilapia

Regarding salinity levels, 30 ppt followed by 15 ppt could significantly increase the level of GH, while there are no significant differences in the levels of ACTH. Regarding vitamin D supplementation, D1 (0.4 mg/kg) and D2 (0.8 mg/kg) increased the levels of GH without any considerable effect on the levels of ACTH as shown in Table 4.

The interaction of VD and salinity showed that at 15 ppt and 0.4 and 0.8 mg of VD and 30 ppt and 0.0, 0.4, and 0.8 mg of VD the GH increased significantly as shown in Table 4. The values of ACTH showed no significant differences due to treatments.

**Table 4:** Qualitative statistical analysis (Mean ± SE) for the effect of vitamin D supplementation under different salinities on hormonal profile (Growth hormone, GH; and Adrenocorticotropic hormone, ACTH) in juvenile red tilapia hybrids

Treatments			GH	ACTH
Main effects				
Salinity level				
0 ppt			0.175$\:\pm\:$0.019^c^	11.33$\:\pm\:$0.323^a^
15 ppt			0.242$\:\pm\:$0.016^b^	11.17$\:\pm\:$0.565^a^
30 ppt			0.280$\:\pm\:$0.009^a^	11.83$\:\pm\:$0.391^a^
Vitamin D				
D0			0.192$\:\pm\:0.028\:$^b^	11.17$\:\pm\:$0.486^a^
D1			0.253$\:\pm\:0.014\:\:$^a^	11.83$\:\pm\:$0.354^a^
D2			0.252$\:\pm\:$0.012^a^	11.33$\:\pm\:$0.464^a^
Two-way ANOVA				
Salinity level			< 0.001	0.574
Vitamin D			< 0.001	0.574
Salinity * VD			< 0.001	0.598
Interaction				
	Salinity	VD		
T1	0 ppt	D0	0.105$\:\pm\:$0.014^e^	11.50$\:\pm\:$0.298^a^
T2		D1	0.205$\:\pm\:$0.014^cd^	11.50$\:\pm\:$0.866^a^
T3		D2	0.215$\:\pm\:$0.014^bcd^	11.00$\:\pm\:$0.577^a^
T4	15 ppt	D0	0.180$\:\pm\:$0.006^d^	10.00$\:\pm\:$0.577^a^
T5		D1	0.290$\:\pm\:$0.006^a^	12.00$\:\pm\:$0.577^a^
T6		D2	0.255$\:\pm\:$0.003^abc^	11.50$\:\pm\:$1.443^a^
T7	30 ppt	D0	0.290$\:\pm\:$0.023^a^	12.00$\:\pm\:$1.115^a^
T8		D1	0.265$\:\pm\:$0.009^ab^	12.00$\:\pm\:$0.577^a^
T9		D2	0.285$\:\pm\:$0.014^a^	11.50$\:\pm\:$0.289^a^

The data is presented as mean $\:\pm\:$ standard error of mean (SEM) in tables.

### Gene expression analysis using real-time PCR

The cDNA obtained from the RNA collected from the tissue of treated red tilapia along with the control group were examined for gene expression analysis of GH and IGF-II in real-time PCR. In this study, GH and IGF-II expressions were observed in all treatment groups. As β-actin is used as the expression reference gene and while comparing the observed expression with the control group, it was found that GH average relative expression was higher in treatment of (0.4 mg VD + 30 ppt salinity), followed by treatment of (0.8 mg VD + 30 ppt salinity) as shown in Table 5. Similar results were obtained for the IGF-II relative gene expression as detected higher in treatment of (0.8 mg VD + 0 ppt salinity) followed by treatment of (0.4 mg VD + 0 ppt salinity). Also, the effect of vitamin D supplementation on IGF-II gene expression was observed under freshwater conditions only, since the salinity (15 and 30 ppt) prevent the IGF-II gene expression even with vitamin D supplementation as shown in Table 6.

**Table 5:** Relative gene expression (RGE) of GH gene (Target Name) in hybrid red tilapia after treatment combination with vitamin D (VD) and salinity

Tr	Salinity	VD	Cт Mean*	RGE
T_1_	0 ppt	D_0_	22.7944	1
T_2_		D_1_	20.4382	18.74326
T_3_		D_2_	22.9677	15.17895
T_4_	15 ppt	D_0_	18.7566	1
T_5_		D_1_	21.0062	2.446128
T_6_		D2	23.3641	2.0558
T_7_	30 ppt	D_0_	17.2105	1
T_8_		D_1_	27.0485	34.25636
T_9_		D_2_	20.4426	25.04065

RGE= Relative gene expression. * Ct Mean: Represents the average of multiple Ct values for the same treatment or biological replicate.

**Table 6:** Relative gene expression (RGE) of IGF-II gene (Target Name) in hybrid red tilapia after treatment combination with vitamin D (V.D) and Salinity

Tr	Salinity		VD	Cт Mean*	RGE
T_1_	0 ppt		D_0_	33.8411	1
T_2_			D_1_	31.8768	14.28471
T_3_			D_2_	33.7656	18.03591
T_4_	15 ppt		D_0_	27.6847	1
T_5_			D_1_	30.7126	1.426223
T_6_			D2	33.7686	0.738823
T_7_	30 ppt		D_0_	19.09	1
T_8_			D_1_	36.9641	0.130507
T_9_			D_2_	29.9928	0.122895

RGE= Relative gene expression. *Ct Mean: Represents the average of multiple Ct values for the same treatment or biological replicate.

## Discussion

The present study revealed that 15 and 30 ppt salinity rearing conditions, vitamin D supplementation (mainly 0.8 mg, followed by 0.4 mg), and their interaction had beneficial effects on the growth performance, feed utilization efficiency, and survival of red tilapia where 0.8 mg of VD at 30 and 15 ppt could achieve the best improvement. This might have been because of the role of salinity on enhanced osmoregulation (as less minimal metabolic energy gets diverted to osmoregulation), reduced metabolic costs, improved digestive efficiency, and improved nutrient absorption^20,37,38^, while vitamin D’s general function supports bone mineralization, muscle development and overall physiological development, including supporting bone and tissue mineralization, enhance tilapia’s swimming ability, basic metabolism, and immune function that directly boost feed efficiency or growth^39–42^. The findings are consistent with Liao and Chang^43^; Sharaf et al.^44^; Sallam et al.^38^; Nassar et al.^20^, findings, who stated that hybrid red tilapia grew quicker and had enhanced growth performance, feed utilization and survival in sea-water and brackish than freshwater. In the same context, the impact of vitamin D in the current research was similar to the findings of Hussein et al.^41^ and Meng^45^ who noted that VD significantly (p˂0.05) increased the average final body-weight, final total length, weight gain weight gain rate and survival of tilapia and vitamin D-treated fish, where fish can synthesize some vitamin D through exposure to sunlight, dietary supplementation is often necessary to ensure optimal health and performance^41,46^. It is critically important for the growth, development, and maintenance of the skeleton’s healthy from birth until death^47,48^.

The current results of feed utilization are consistent with Sharaf et al.^44^; Nassar et al.^20^ who described that sea water (26 ppt) increased the ADG and SGR of red tilapia significantly while brackish (13 ppt) and sea water (26 ppt) enhanced the values of FCR and PER. Salinity levels can significantly impact the growth performance of red tilapia (*O. mossambicus x O. niloticus*), particularly in terms of average daily gain (ADG), specific growth rate (SGR), protein efficiency ratio (PER) and feed conversion ratio (FCR)^20,38^. In the same context, the impact of vitamin D on the feed utilization was similar to the findings of Inayat et al.^40^; Hussein et al.^41^ who reported that VD enhanced ADG, FCR and SGR of tilapia and *Labeo rohita* respectively.

In fish, growth hormone (GH) is created by the adenohypophysis, and functions as multipotent hormone regulator of different physiological processes^49^. In the present study, vitamin D and salinity have a beneficial impact on the growth hormone level of hybrid red tilapia. The treatments T_5_ (15 ppt and 0.4 mg of VD), T_6_ (15 ppt and 0.8 mg of VD), T_7_ (30 ppt and 0.0 mg of VD), T_8_ (30 ppt and 0.4 mg of VD) and T_9_ (30 ppt and 0.8 mg of VD) enhanced the level of growth hormone and achieved the peak levels. The obtained results may be due to salinity significantly influencing growth hormone (GH) levels in tilapia, affecting their growth performance and physiological responses^50^. Studies have demonstrated that tilapia raised in seawater exhibit significantly higher levels of GH in their pituitaries compared to those reared in freshwater. For instance, tilapia raised in seawater for 7 months contained more GH than those in freshwater, indicating that salinity enhances GH cell activity and potentially promotes growth^50,51^. Moreover, the role of VD in the raising of GH levels may be due to its significant role in fish’s growth and development, particularly through its influence on growth hormone (GH) and metabolic processes^52^. Sufficient VD is essential for optimal GH levels and overall growth performance in tilapia. VD deficiency can disrupt GH signaling and lead to metabolic disorders, while adequate VD supplementation enhances growth metrics and reproductive health^23,47,50^. Also, Studies indicate that dietary supplementation of VD enhances growth performance in juvenile gilthead seabream, positively influencing GH levels and overall growth metrics. Thus, adequate VD is crucial for optimal growth in this species^53^. Understanding the correlation between VD and growth hormone is crucial for developing effective aquaculture practices and improving tilapia production.

In the present study, there are non-significant impacts of vitamin D and salinity levels on the level of ACTH. Those findings are agreed with Seale et al.^54^ who noted that in tilapia, the fresh and sea - water didn’t significantly affect the level of ACTH, while the effect of VD on adrenocorticotropic hormone (ACTH) levels in tilapia has not been directly addressed in the available literature. The effect of salinity on adrenocorticotropic hormone (ACTH) levels in tilapia has not been extensively studied, but exposure to different salinity levels can induce stress in tilapia, which may lead to alterations in the secretion of stress hormones, including ACTH^50,55^. Limited studies were done imply how salinity affects ACTH and/or growth hormone (GH) levels in tilapia. Increased GH levels in response to higher salinity could suggest a compensatory mechanism to promote growth and osmoregulation, which might also influence ACTH dynamics indirectly through stress responses^51,55^. While the direct relationship between VD and ACTH levels in tilapia is not explicitly studied, VD’s role in overall hormonal balance suggests it could influence stress-related hormones like ACTH indirectly^23,41^. If VD supplementation leads to better overall health and reduced stress in tilapia, it may result in lower ACTH levels, as chronic stress is known to elevate ACTH and cortisol^56^. Further research is needed to clarify this relationship and understand the mechanisms involved.

These obtained results revealed that vitamin D supplementation enhanced the expression of growth hormone genes in high salinity as VD has immunomodulatory effects. Slightly similar results were observed as correlated to the final weight and weight gain. Since the highest final weight was achieved in treatment of (0.8 mg vitamin D and 30 ppt salinity) followed by the treatment of (0.8 mg vitamin D and 15 ppt salinity) supporting the suggestion of vitamin D enhanced the weight under salinity conditions. Also, for the plasma concentration of GH, the highest concentration was observed in the treatment of (0.4 mg VD) under both conditions of salinity (15 and 30 ppt). Results of GH plasma concentration revealed the effect of combination between salinity and vitamin D supplantation, that the vitamin D enhanced the performance under salinity by increasing the regulation of GH gene expression which is reflected by increased GH plasma as compared to the control group and VD supplementation under freshwater conditions.

The GH and IGF genes have critical importance, which are fundamental in the neuroendocrine regulation of fish growth and development. Those functions may extend into immune responses, osmoregulation, and reproductive processes, making it a multifaceted gene system essential for fish physiology and aquaculture productivity^57–59^. In the current study, vitamin D supplementation under different salinities impacts the expression of growth hormone (GH) and insulin-like growth factor (IGF) genes in juvenile hybrid red tilapia by modulating the endocrine axis regulating growth and osmoregulation. Vitamin D influences hormone secretion and gene expression in fish, affecting not only calcium and phosphorus homeostasis but also growth through the GH/IGF axis. Supplementing VD can raise circulating hormone levels, which supports development and metabolic activity under varying environmental conditions^60–62^. Exposure to varying salinity environments distinctly alters the GH/IGF axis. Studies on Mozambique tilapia indicate that fish reared in different salinities display unique patterns of GH and IGF gene expression and often show accelerated growth rates. Generally, higher pituitary GH mRNA and changes in IGF-I, IGF-II, and growth hormone gene expression in muscle and liver are observed. These adjustments are closely linked to enhanced osmoregulatory and growth capacity^62–64^. Although direct studies on VD and salinity effects in hybrid red tilapia are scarce, evidence suggests that VD enhances the capacity of the GH/IGF system to respond to environmental salinity challenges. This results in increased GH and IGF gene expression, facilitating growth and osmoregulatory adaptation as the fish acclimate to freshwater or saline habitats^62^. In environments with varying salinity, fish need to adjust their internal salt concentrations to survive, here the role of IGF affect to maintain the balance of water and salts in their bodies through osmoregulation process^65^. From the above findings, VD supplementation acts synergistically with environmental salinity cues to optimize the GH/IGF endocrine axis, benefitting juvenile hybrid red tilapia in terms of growth and adaptability to changing water conditions.

## Conclusion

These findings highlight the complex physiological adaptations of red tilapia hybrid juveniles to different salinity levels and the beneficial effects of vitamin D supplementation. This provides valuable insights for optimizing culture practices in Egypt for the promising hybrid red tilapia amid climate change and freshwater scarcity. Specifically, administering 0.8 mg of vitamin D followed by 0.4 mg under varying salinity conditions may enhance growth performance, feed efficiency, growth hormone levels, and the expression of GH and IGF genes. Additionally, during culture, maintaining salinity levels at 30 ppt followed by 15 ppt appears to improve fish performance compared to freshwater culture.

## Data Availability

Data will be made available on request from the corresponding author due to privacy.
